# Engineering the Surface/Interface Structures of Titanium Dioxide Micro and Nano Architectures towards Environmental and Electrochemical Applications

**DOI:** 10.3390/nano7110382

**Published:** 2017-11-09

**Authors:** Xiaoliang Wang, Yanyan Zhao, Kristian Mølhave, Hongyu Sun

**Affiliations:** 1 College of Science, Hebei University of Science and Technology, Shijiazhuang 050018, China; wxlsr@126.com; 2 Department of Chemistry Boston College Merkert Chemistry Center, 2609 Beacon St., Chestnut Hill, MA 02467, USA; zhaogh@bc.edu; 3 Department of Micro- and Nanotechnology, Technical University of Denmark, Kongens Lyngby 2800, Denmark

**Keywords:** titanium dioxide, crystal structure, surface/interface structure, photocatalysis, lithium/sodium ion batteries, Li–S batteries, phase stability

## Abstract

Titanium dioxide (TiO_2_) materials have been intensively studied in the past years because of many varied applications. This mini review article focuses on TiO_2_ micro and nano architectures with the prevalent crystal structures (anatase, rutile, brookite, and TiO_2_(B)), and summarizes the major advances in the surface and interface engineering and applications in environmental and electrochemical applications. We analyze the advantages of surface/interface engineered TiO_2_ micro and nano structures, and present the principles and growth mechanisms of TiO_2_ nanostructures via different strategies, with an emphasis on rational control of the surface and interface structures. We further discuss the applications of TiO_2_ micro and nano architectures in photocatalysis, lithium/sodium ion batteries, and Li–S batteries. Throughout the discussion, the relationship between the device performance and the surface/interface structures of TiO_2_ micro and nano structures will be highlighted. Then, we discuss the phase transitions of TiO_2_ nanostructures and possible strategies of improving the phase stability. The review concludes with a perspective on the current challenges and future research directions.

## 1. Introduction

Environment and energy are important factors, which affect the sustainable development of the society. Clean energy techniques and environmental treatment solutions based on advanced nanomaterials, which are earth abundant and environmentally compatible show the potential to solve the crisis. Titanium dioxide (TiO_2_) is such a material that satisfies the criteria [1,2]. As an important and widely used wide bandgap (3.0–3.2 eV) oxide semiconductor, TiO_2_ shows unique physical and chemical properties [3]. The applications of TiO_2_ materials range from conventional fields (cosmetic, paint, pigment, etc.) to functional devices, such as photo- or electrocatalysis, photoelectrochemical or photovoltaic cells, lithium/sodium ion batteries, Li–S batteries, and biotechnological applications [4,5,6,7,8,9,10,11,12,13].

**Figure 1:**
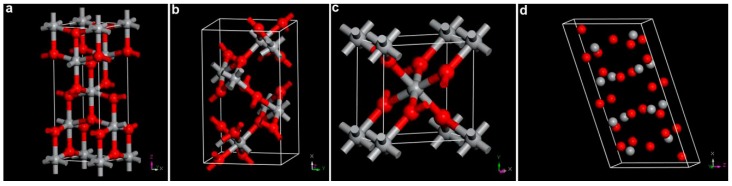
Crystal structures of typical TiO_2_ polymorphs: (**a**) rutile; (**b**) brookite; (**c**) anatase; and (**d**) TiO_2_(B). Gray and red spheres are Ti^4+^ and O^2−^ ions, respectively.

There are at least 11 reported bulk or nanocrystalline phases of TiO_2_. In nature, TiO_2_ forms four main phases: rutile, anatase, brookite, and TiO_2_(B). The crystal models of the four structures are illustrated in Figure 1. All of these TiO_2_ phases can be seen as constructed by Ti–O octahedral units. The main structural difference is the connecting ways of the basic Ti–O octahedral repetitive units. For instance, octahedra shares two, three, and four edges in rutile, brookite, and anatase phase, respectively. In TiO_2_(B) phase, the Ti–O octahedral connection is similar to the anatase one, but with a different arrangement that shows layer character [14]. Under the condition of normal temperature and atmospheric pressure, the relative stability of bulk phase is rutile > brookite > anatase > TiO_2_(B) [15]. However, this stability order can be changed by ambient condition and sample properties (particle size, morphology, surface state, etc.). The four TiO_2_ phases can be distinguished by using diffraction, Raman spectroscopy, or electrochemical techniques. Due to the structural difference, these TiO_2_ phases each have their specific applications. Therefore, it is important to study the phase transformation among different phases and develop methods to improve the phase stability [16,17,18]. 

For a given TiO_2_ phase, size and morphology play important roles in the energy conversion and storage. In this regard, TiO_2_ nanostructures with well controlled geometric dimension and morphology, such as nanoflowers [19,20,21], inverse opal- [22,23,24,25,26,27], urchin- [28,29,30], and dandelion-like [31,32,33] structures, have been successfully explored. Besides those geometric parameters, the surface and interface structures are also responsible for the applications mentioned above [12,13,34]. Photo- or electrocatalysis requires the effective adsorption and desorption of reactant molecules/ions and intermediate products on the surface of TiO_2_ photocatalysts [35,36,37,38]. The ions transportation is occurred across the surface or interface of TiO_2_ electrodes during the continuous charging and discharging processes in lithium/sodium ion batteries [39]. Adjusting the interaction between sulfur cathodes and the surface of TiO_2_ host is important to improve the cycle stability of Li–S batteries with a higher capacity than those of lithium ion batteries [40]. Therefore, engineering the surface/interface structures of TiO_2_ crystals is not only fundamentally important for studying the essential interaction between molecules or ions and TiO_2_, but is also valuable to the technical applications [41,42].

In this paper, we summarize the most recent progress in engineering the surface/interface structures of TiO_2_ micro and nano structures for the applications in environment and electrochemistry. The article is organized as follows: Section 2 analyzes the benefits of surface/interface engineered TiO_2_ micro and nano structures; Section 3 reviews the main strategies used for surface/interface engineering in TiO_2_ materials; Section 4 evaluates the advantages and different application of surface/interface engineering in the context of photocatalytic degradation of organic contaminants, water-splitting, CO_2_ reduction, antimicrobial and self-cleaning, electrodes for lithium/sodium ion batteries, and Li–S batteries; Section 5 discusses the phase stability of typical TiO_2_ structures, and the possible routes to improve the stability; and, finally, we will provide our perspective on the current challenges and important research directions in the future.

## 2. Advantages of Surface/Interface Engineered TiO_2_ Micro and Nano Structures 

When compared to the TiO_2_ materials in bulk form or other nanostructures, the surface/interface engineered TiO_2_ micro and nano structures are promising to transcend the difficulties in photocatalysis and energy storage applications. The benefits of TiO_2_ materials with well controlled surface and interface structures are briefly summarized as follows.

(1) Large specific surface area. The surface area of TiO_2_ materials plays an important role in their photocatalytic activity and ion storage ability. Firstly, large surface area can increase the contact area with electrolyte, and thus the amount of active reaction sites for photocatalytic applications. Secondly, the high surface area of TiO_2_ electrodes is also favorable for the storing more ions.

(2) Tunable band structure and bandgap. The electronic structure of TiO_2_ materials can be tuned by engineering surface and interface configurations. Due to the intrinsic limitations of the wide bandgap in bulk form, the practical use of pristine TiO_2_ materials in the fields of photocatalysis is hampered. Only ultraviolet (UV) light (<5% of the full solar spectrum) can activate the TiO_2_ photocatalysts. By employing surface modification via defect generation, doping, or interface formation, the band structure and the bandgap value of various TiO_2_ materials can be adjusted, making it possible to achieve efficient and durable visible light photocatalysis [5,6,7,8,9,10,11,12,13].

(3) Improved electronic and ionic conductivity. The modulated band structure and bandgap in TiO_2_ materials generate additional state within the forbidden band, which facilitates the fast transport of ionic and electronic species, and are important for the rapid migration, transport, and recombination of carriers for catalysis, and high rate battery applications.

(4) Optimized interaction between reactant molecules/ions, intermediate products, and the surface of TiO_2_ materials. The binding of species on the engineered TiO_2_ surface can be adjusted. It is important to improve the catalytic activity and selectivity, and promote electrochemical performance for novel energy storage device, such as Li–S batteries.

## 3. Strategies in Surface/Interface Engineering of TiO_2_ Micro and Nano Structures 

The above discussion shows that surface and interface structures in TiO_2_ materials are related to the electronic/optical properties and thus diverse applications ranging from energy to environment. So far, different methods have been proposed to control the surface and interface configurations for TiO_2_ micro and nano structures [43,44,45]. Among the methods, a primary classification can be made by distinguishing physical and chemical methods, which are based on top-down and bottom-up approaches, respectively. There are several excellent reviews describing the specific synthesis methods (such as self-assembly, template, hydrothermal, solvothermal, annealing, electrochemical method, etc.) to control the surface/interface structures [5,34,46]. In this paper, we avoid describing the different synthesis methods, but discuss fundamental strategies, including one-step (sometimes called in-situ) methods, post treatment, and theoretical guidance, those are used to engineer the surface/interface structures. 

### 3.1. One-Step Approach

In order to modify the surface/interface structures via the one-step approach, understanding the nucleation and further growth is essential. Up to now, solution-based and vapor-based approaches have been developed to control the nucleation and growth, and different mechanisms including vapor−liquid−solid, orientation attachment, Ostwald ripening, surfactant-controlled, and growth by surface reaction limitation have been proposed, which have been reviewed elsewhere [5,34,46]. 

**Figure 2:**
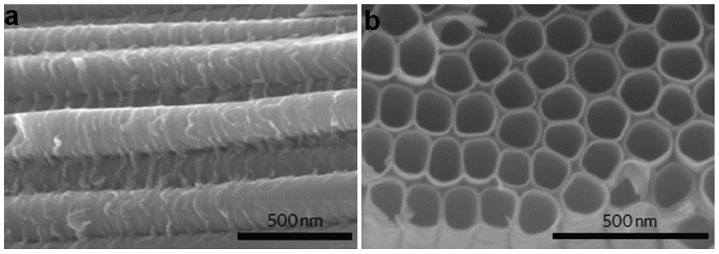
Engineering the surface/interface structures in TiO_2_ materials via one step approach. (**a**) Cross section and (**b**) front view scanning electron microscopy (SEM) images of amorphous TiO_2_ nanotube arrays fabricated by anodic oxidation. Reproduced with permission from [47], Copyright Nature Publishing Group, 2010.

Richter et al. [47] fabricated aligned TiO_2_ nanotube arrays by the oxidation of a titanium foil in hydrofluoric acid solution (0.5–3.5 wt %). Electron microscopy images showed that the tubes were open on the tops and were closed on the bottoms. The average tube diameter grew with the increasing of voltage, while the length was independent on reaction time. Field-enhanced void structure was responsible for the tube formation. By suitable choice of the pH value, electrolytes and the Ti sources, the geometry and composition of the nanotube arrays can be controlled more precisely (Figure 2).

Penn et al. [48] proposed that some TiO_2_ nanostructures could be formed in solution through the route of oriented attachment, where the merger of nanocrystals is based on orientations of each nanoscale crystal to form single crystalline structure. Experiment and simulations showed that the driving force of an oriented attachment was the reduction of the total surface energy contributed by the removal of certain crystal facets with a high surface energy. The kinetic behaviors of the oriented attachment growth was directly related to the solution properties and reaction temperature. Therefore, it is possible to control the surface/interface properties of the final TiO_2_ nanostructures by modifying the crystal facets of the pristine nanocrystals, as well as solution viscosity and others.

### 3.2. Post Treatment Routes

Based on the well-established top-down and bottom-up strategies, the synthesis of TiO_2_ micro and nano structures with controllable parameters, such as size, morphology, composition, as well as assembly, can be achieved. Those TiO_2_ materials with well-defined geometry and chemistry provide abundant possibilities to further tune the atomic scale structures. Therefore, different post-treatment techniques, including thermal annealing, laser irradiation, electrochemical cycling, and solution reaction, have been developed to yield TiO_2_ materials with modified surface and interface structures [49,50,51,52,53,54,55]. 

**Figure 3:**
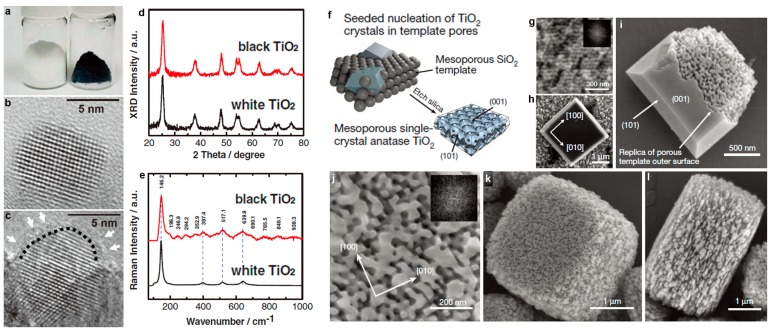
Post treatment route to tune the surface/interface structures in TiO_2_ materials. (**a**) A photo comparing unmodified white and disorder-engineered black TiO_2_ nanocrystals; (**b**,**c**) High-resolution transmission electron microscopy (HRTEM) images of TiO_2_ nanocrystals before and after hydrogenation, respectively. In (**c**), a short dashed curve is applied to outline a portion of the interface between the crystalline core and the disordered outer layer (marked by white arrows) of black TiO_2_; (**d**,**e**) X-ray Diffraction (XRD) and Raman spectra of the white and black TiO_2_ nanocrystals (reprinted from [49] with permission, Copyright American Association for the Advancement of Science, 2011). (**f**) Schematic and (**g**–**l**) electron microscopy images of mesoporous single-crystal nucleation and growth within a mesoporous template. (**g**) Pristine silica template made up of quasi-close-packed silica beads; (**h**) non-porous truncated bipyramidal TiO_2_ crystal; (**i**) template-nucleated variant of the crystal type shown in (**h**); (**j**) replication of the mesoscale pore structure within the templated region; (**k**,**l**) fully mesoporous TiO_2_ crystals grown by seeded nucleation in the bulk of the silica template. (Reproduced with permission from [44], Copyright Nature Publishing Group, 2013).

By employing high pressure (~20 bar) hydrogen annealing treatment, Chen et al. [49] successfully converted the pristine white TiO_2_ nanoparticles into black hydrogenated particles (Figure 3a–e). The color change indicated that the optical absorption properties had been modified through the treatment. Further structural characterizations showed that the obtained black TiO_2_ nanoparticles possessed crystalline core/amorphous shell structure. The surface layer with disordered feature was due to hydrogen dopant, leading to the formation of hydrogen related bonds (such as Ti–H, O–H). Such hydrogen dopant induced surface modification also generates midgap stated, and thus makes the color of the sample as black. Similar to the case of hydrogen treatment, annealing in oxygen deficient atmosphere also results in the effective modification of the surface/interface structures. Huang et al. [50] reported a facile solution reaction, followed by nickel ions assisted ethylene thermolysis to synthesize rutile TiO_2_ nanoparticles. The surface of each nanoparticle was etched to form pits with an average size of 2–5 nm (quantum pits). Based on the characterizations, they proposed a possible formation mechanism for the quantum pits. Thanks to the ethylene thermolysis during annealing, a carbon layer was formed on the surface of TiO_2_ nanoparticles. The carbon layer then reacted with trace Cl_2_ in the chamber, inducing the etching of TiO_2_ locally based on the reaction: TiO_2_ + 2C + 2Cl_2_ ↔ TiCl_4_ + 2CO. The microstructure of the rutile TiO_2_ nanoparticles is very unique. The abundant quantum-sized pits on the surface generate defect structures and unsaturated bonds, which are important for improving the conductivity and ion storage. Laser irradiation in liquids is also an useful method to modify the surface and interface of different TiO_2_ nanostructures [56,57]. During the experiment, laser wavelength, laser energy, irradiation time, and the solution that is employed can be chosen to control the surface structure [58], bandgap, and even phase transformation [59]. In a recent work shown by Filice et al. [58], under-coordinated Ti ions and distorted lattice were formed on the surface of TiO_2_ nanoparticles upon laser irradiation, which were important in the modification of the physical and chemical properties. Recently, electrochemical cycling in different mediums (aqueous, organic solution, and ionic liquids) have been used to modify the surface composition, as well as microstructure of TiO_2_ materials. The results show that the surface defect structures, especially oxygen vacancies, and their amount can be controlled by adjusting the electrochemical conditions.

Template assistance is also effective to control the surface/interface of TiO_2_ micro and nano configurations. Crossland et al. [44] developed a mesoporous single-crystal anatase TiO_2_ based on seed-mediated nucleation and growth inside of a mesoporous template (Figure 3f). In a typical process, silica template was firstly seeded by pre-treatment in a solution of TiCl_4_ at 70 °C for 60 min. The anatase TiO_2_ mesoporous single-crystal was obtained via hydrothermal reaction of TiF_4_, with the addition of hydrofluoric acid and pre-treated silica template. The template was then removed by adding aqueous NaOH solution to recover the mesoporous TiO_2_ crystals. The final product reveals facet truncated bipyramidal crystals with external symmetry matching that of the homogeneously nucleated bulk crystals, whose mesoscale structure is a negative replica of the silica template. Compared to the conventional TiO_2_ nanocrystalline, the TiO_2_ mesoporous single-crystal shows a higher conductivity and electron mobility.

### 3.3. Theoretical Guidance

With the rapid development of modern calculation and simulation, computational material methods based on diverse scale, such as finite element, large scale molecular dynamics (MD) simulation, and density functional theory (DFT) are becoming more and more powerful to provide fundamental insights into experimental results, and more importantly, design and predict the performance of novel functional materials. With the assistance of theoretical methods, it is possible to understand the nucleation, growth, surface properties in liquid and gas environment, which is important to realize controllable synthesis and optimize physical/chemical properties of the nanomaterials [60,61,62]. 

The equilibrium morphology of a crystal is given by the standard Wulff construction, which depends on the surface/interface properties. Barnard and Curtiss investigated the effects of surface chemistry on the morphology of TiO_2_ nanoparticles by using a thermodynamic model based on surface free energies and surface tensions obtained from DFT calculations. In the condition of hydrated, hydrogen-rich, and hydrogenated surfaces, the shape of anatase and rutile nanoparticles vary little, however, in the case of hydrogen-poor and oxygenated surfaces, the anatase and rutile nanocrystals become elongated. The results show that the exposed facets of the TiO_2_ nanocrystals can be controlled through modifying the surface acid-base chemistry. 

**Figure 4:**
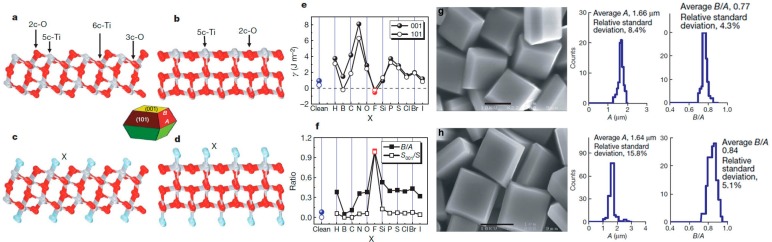
Theoretical calculation guides the modification of surface/interface structures. (**a**–**f**) Slab models and calculated surface energies of anatase TiO_2_ (001) and (101) surfaces. (**a**,**b**) Unrelaxed, clean (001) and (101) surfaces; (**c**,**d**) Unrelaxed (001) and (101) surfaces surrounded by adsorbate *X* atoms; (**e**) Calculated energies of the (001) and (101) surfaces surrounded by *X* atoms; and, (**f**) Plots of the optimized value of B/A and percentage of {001} facets for anatase single crystals with various adsorbate atoms *X*. Here, the parameters of *A* and *B* are the lengths of the side of the bipyramid and the side of the square {001} “truncation” facets (see the geometric model). The value of B/A describes the area ratio of reactive {001} facets to the total surface. (**g**,**h**) SEM images and statistical data for the size and truncation degree of anatase single crystals (Reproduced with permission from [43], Copyright Nature Publishing Group, 2008).

Besides the acid-base condition, heterogeneous atoms or surfactant adsorption can also affect the surface and interface structures. Based on DFT calculations, Yang et al. [43] systematically studied the adsorption of a wide range of heterogeneous non-metallic atoms *X* (*X* = H, B, C, N, O, F, Si, P, S, Cl, Br, or I) on {001} and {101} facets of anatase TiO_2_ crystals (Figure 4). The results show that the adsorption of F atoms not only decreases the surface energy for both the (001) and (101) surfaces, but also results in the fact that (001) surfaces are more stable than (101) surfaces, i.e., the F adsorption is favorable for the formation of (001) facets in anatase TiO_2_. The theoretical results inspire intense studies on the surface structure control of TiO_2_ crystals. Experimentally, a mixture containing titanium tetrafluoride (TiF_4_) aqueous solution and hydrofluoric acid was hydrothermally reacted, to generate the truncated anatase bipyramids, and anatase TiO_2_ single crystals with a high percentage of {001} facets were obtained.

## 4. Applications of Surface/Interface Engineered TiO_2_ Micro and Nano Structures

Surface and interface structures of TiO_2_ materials play important roles in multiple physical/chemical processes. Herein, we will highlight the recent progress in the research activities on the surface/interface engineered TiO_2_ micro and nano structures that are used for photocatalysis (including photocatalytic degradation of organic contaminants, photocatalytic hydrogen evolution, photocatalytic CO_2_ reduction, antimicrobial, and self-cleaning), lithium/sodium ion batteries, and Li–S batteries.

### 4.1. Photocatalysis

**Figure 5:**
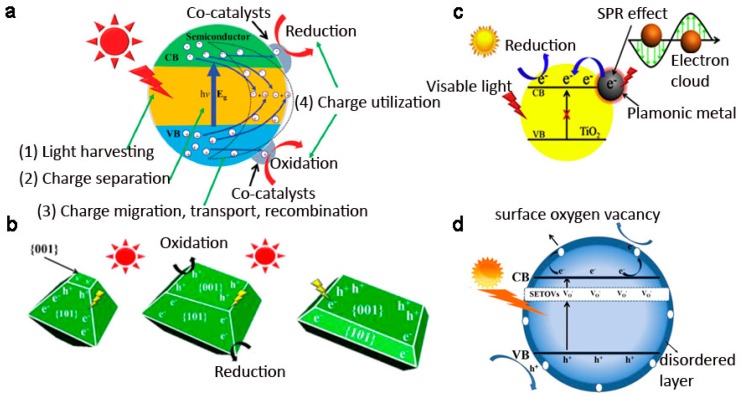
(**a**) Different stages in heterogeneous photocatalysis (Reproduced with permission from [63], Copyright The Royal Society of Chemistry, 2016); surface/interface engineered TiO_2_ structures for photocatalytic improvement: (**b**) crystallographic plane tuning (Reproduced with permission from [64], Copyright American Chemical Society, 2014), (**c**) defects engineering (Reproduced with permission from [65], Copyright Elsevier B.V., 2016), and (**d**) creating interfaces in TiO_2_ nanostructures (Reproduced with permission from [66], Copyright Elsevier B.V., 2017).

There are four main steps involved in heterogeneous photocatalysis process (Figure 5a): (1) light absorption; (2) the generation and separation of photoexcited electrons and holes; (3) the migration, transport, and recombination of carriers; and, (4) surface catalytic reduction and oxidation reactions. The overall photocatalysis efficiency is strongly dependent on the cumulative effects of these four consecutive steps. Among different photocatalyst materials, TiO_2_ is considered to be a remarkable photocatalyst due to the notable merits such as nontoxicity, biological compatibility, and universality. Since the photocatalytic reaction is a surface or interface sensitive process, control of the surface/interface structures in TiO_2_ materials provides a possible way to improve the light absorption and visible light usage, and facilitate the carrier separation, resulting in enhanced photocatalytic properties. Many attempts have been carried out to modify the surface or interface structures of TiO_2_ materials, such as exposed crystallographic plane tuning, defect engineering, interface construction, and so on (Figure 5b–d). In the following, we will discuss the effects of those surface/interface modifications on the photocatalytic degradation of organic contaminants, photocatalytic hydrogen evolution, and photocatalytic CO_2_ reduction. Other environmental applications such as antimicrobial and self-cleaning are also briefly discussed. 

#### 4.1.1. Photocatalytic Degradation of Organic Contaminants

With a rapidly growing world population and expanding industrialization, the development of new materials, techniques, and devices those can provide safe water and air is important to the societal sustainability. Semiconductor photocatalysis has been utilized as an ideal way to degrade various organic contaminants in water and air. 

Edy et al. [67] synthesized free-standing TiO_2_ nanosheets with different thickness via atomic layer deposition on a dissolvable sacrificial polymer layer. The photocatalytic performance was evaluated for photocatalytic degradation of methyl orange under UV light irradiation. The photocatalytic activity increases with increasing the thickness, which may be due to the existence of Ti^3+^ defect and locally ordered domain structures in the amorphous nanosheets. TiO_2_ nanostructures with exposed highly reactive facets, for example, anatase TiO_2_ nanosheets with {001} facets, are desirable for the photocatalytic enhancement. Those thin nanosheets are prone to aggregate during the practical usage, which results in the loss of photocatalytic activity. Assembly the individual nanostructure into hierarchical architecture can not only suppress the aggregation of micro/nanoscale building blocks, but also increase specific surface area and the amount of active reaction sites, and reduce the diffusion barrier. We synthesized anatase TiO_2_ hollow microspheres assembled with high-energy {001} facets via a facile one-pot hydrothermal method [68]. The percentage of exposed {001} facets on the microspheres was estimated to be about 60%. The photocatalytic ability was evaluated by photodegradation of methylene blue under UV light. The photocatalytic degradation reaction follows pseudo-first-order kinetics among the studied samples. The apparent photochemical degradation rate constant for the hierarchical TiO_2_ structures is 4.07 × 10^−2^ min^−1^, which is faster than that of control samples (TiO_2_ powders (Degussa, P25), 3.11 × 10^−2^ min^−1^; porous TiO_2_ powders, 2.76 × 10^−2^ min^−1^; the etched TiO_2_ spheres, 2.17 × 10^−2^ min^−1^; the irregular TiO_2_ product, 0.86 × 10^−2^ min^−1^). The good photocatalytic activity of the hierarchical TiO_2_ structures is associated with the hollow structures with bimodal mesopore size distribution and relatively large Brunauer–Emmett–Teller (BET) surface areas. Xiang et al. [69] synthesized a kind of hierarchical flower-like TiO_2_ superstructures by alcohothermal treatment method. The superstructures consisted of anatase TiO_2_ nanosheets with 87% exposed (001) facets. Photocatalytic oxidative decomposition of acetone was evaluated in air under UV light. The results show that the photocatalytic activity of the flower-like TiO_2_ superstructures was better than that of P25 and tubular shaped TiO_2_ particles. The synergetic effect of highly exposed (001) facets hierarchically porous structure, and the increased light-harvesting capability is responsible for the enhanced photocatalytic ability.

Besides the exposed high energy facets, the introduction of suitable defect structures in TiO_2_ materials can obviously influence the light absorption and the separation of photogenerated electron-hole pairs [70,71,72]. Cao et al. [70] fabricated mesoporous black TiO_2_ spheres with high crystallinity by a facile evaporation-induced self-assembly method combined with mild calcinations after an in-situ hydrogenation under an argon atmosphere. The results indicated that the prepared sample was uniform mesoporous black spheres with Ti^3+^ and N co-doping. The visible-light-driven photocatalytic degradation ratio of methyl orange was up to 96%, which was several times higher than that of pristine TiO_2_ material. The excellent photocatalytic activity was due to Ti^3+^ and N doping, which resulted in high visible light utilization and enhanced separation of photogenerated charge carriers, and the mesoporous network structures. 

Generating interface structures by depositing plasmonic-metal nanostructures (Ag, Pt, Au, etc.) on TiO_2_ materials can increase the generation rate of energetic charge carriers and result in a higher probability of redox reactions [73,74,75,76]. By using successive ion layer adsorption and reaction, Shuang et al. [73] synthesized TiO_2_ nanopillar arrays with both Au and Pt nanoparticles (~4 nm) decoration. Due to the electron-sink function of Pt and surface plasmon resonance of Au nanoparticles, the charge separation of photoexcited TiO_2_ was improved. The obtained Au/Pt nanoparticles decorated TiO_2_ nanopillar arrays showed a much higher visible and UV light absorption response, which lead to remarkably enhanced photocatalytic activities in the degradation of methyl orange. 

#### 4.1.2. Photocatalytic Hydrogen Evolution

Hydrogen energy is one of the most promising green fuels. Since the first discovery of photoelectrochemical water splitting by Fujishima and Honda in 1972, hydrogen production directly from water and sunlight on semiconductor materials has been intensively investigated [3]. Although numerous semiconductor materials have been explored as photocatalysts to produce hydrogen, TiO_2_ remains one of the most studied materials for photocatalytic H_2_ evolution due to the main merits of nontoxic and chemical stability. 

Highly reactive exposed facets of TiO_2_ nanostructures are related to the photocatalytic activity enhancement. For example, Wu et al. [77] synthesized mesoporous rutile TiO_2_ single crystal with wholly exposed {111} facets by a seeded-template method. Fluoride ions in the solution played an important role in stabilizing the high energy facet {111} of rutile TiO_2_. The ratios of exposed {110} and {111} facets can be controlled by tuning the concentration of fluoride ions. The mesoporous single crystal rutile TiO_2_ with wholly exposed {111} reactive facets exhibited a greatly enhanced photocatalytic hydrogen generation. Zhang et al. [78] demonstrated that the TiO_2_ single crystal with a novel four-truncated-bipyramid morphology could be synthesized by a facile hydrothermal reaction. The resultant photocatalyst exhibited excellent hydrogen evolution activity from ethanol-water solution. The exposure of both high-energy {001} oxidative and low-energy {101} reductive facets in an optimal ratio are thought to be the key factors for the high photocatalytic activity. In another example, anatase TiO_2_ nanoplates with exposed (001) facet were converted from the NH_4_TiOF_3_ nanoplates [79]. The obtained compact TiO_2_ nanoplates exhibited a high H_2_-production rate of 13 mmol·h^−1^·g^−1^ with a H_2_-production quantum efficiency of 0.93% at 365 nm. 

The influence of defect structures in TiO_2_ materials on photocatalytic H_2_-evolution is complicated. For one thing, the defects could introduce additional states in the band gap, which cause the recombination of carriers and the weakening of carriers’ oxidation and reduction capacities [80,81,82,83]. For another, subtly generating specific defects will facilitate the separating of the carriers. Recently, Wu et al. [80] prepared yellow TiO_2_ nanoparticles with ultra-small size of ~3 nm. Simulated solar light driven catalytic experiments showed that the evolved H_2_ for the yellow TiO_2_ was ~48.4 μmol·h^−1^·g^−1^, which was ~3.7 fold when comparing to that of the normal TiO_2_ (~13.1 μmol·h^−1^·g^−1^) at the same experimental conditions. It is suggested that the significantly improved H_2_-evolution activity can be attributed to the coexistence of titanium vacancies (acceptor) and titanium interstitials (donor) in the TiO_2_ materials, which is beneficial for the spontaneous separation of photo-generated charge-carriers. When compared to the complex steps that are required to accurately control of the defects, the passivation of the defect states with elemental doping would be more direct. Recent works show that Mg doping could eliminate the intrinsic deep defect states and weaken the shallow defect states in TiO_2_ materials [83]. The result was confirmed by the transient infrared absorption-excitation energy scanning spectroscopic measurement. The photocatalytic over-all water splitting measurements showed the H_2_ and O_2_ evolution rates can be as high as 850 and 425 μmol·h^−1^·g^−1^ under Air Mass (AM) 1.5 G irradiation and the apparent quantum efficiency of 19.4% was achieved under 350 nm light irradiation.

Rational creating hetero- or homo-interfaces can achieve high-performance photocatalytic hydrogen evolution. When compared to the pure crystalline and amorphous TiO_2_ film, high electron concentration and mobility can be concurrently obtained at the homo-interface between crystalline and amorphous layers in a bilayer TiO_2_ thin film. Therefore, extraordinary properties could be explored in well-designed interfaces with homogeneous chemical composition. By creating a crystalline Ti^3+^ core/amorphous Ti^4+^ shell structure, Yang et al. [84] successfully activated rutile TiO_2_ material with efficient photocatalytic hydrogen evolution properties. The average hydrogen evolution rate was enhanced from 1.7 for pure TiO_2_ to 268.3 μmol·h^−1^ for TiO_2_ with homointerface structures. The origin of the activation was attributed to the regulated the transport behaviors of holes and electrons from the bulk of a particle to the surface by suppressing the transport of electrons in the conduction band and facilitating the transport of holes in the valence band. In addition, hetero-interfaces between TiO_2_ materials and other semiconductor or metal nanostructures, including carbon, Si, NiO, ZnS, CdS, MoS_2_, MoC_2_, layered double hydroxides, and plasmonic metals, has been extensively investigated [85,86,87,88,89,90,91]. As an example, Wu et al. [85] reported that anisotropic TiO_2_ overgrowth on Au nanorods could be obtained by selective spatial assembly and subsequent hydrolysis. Plasmon-enhanced H_2_ evolution under visible/near-infrared light irradiation has been demonstrated. The Au nanorod-TiO_2_ interface with the Au nanorod side exposed, as a Schottky junction, can filter out surface plasmon resonance hot electrons from the Au nanorod, which is crucial to boosting the H_2_ evolution performance. 

#### 4.1.3. Photocatalytic CO_2_ Reduction

Due to the increasing consumption of conventional fossil fuels, the concentration of greenhouse gas, especially CO_2_, steadily grows over years. Solar-light-driven reduction of CO_2_ to useful chemical fuels (such as CH_4_, HCO_2_H, CH_2_O, and CH_3_OH) is a promising solution for the serious environmental and energy problems. In the process of photocatalytic CO_2_ reduction, typical steps including adsorption of CO_2_, generation of electron-hole pair, separation and migration of electron-hole pair, and the reduction of CO_2_ are involved. Since CO_2_ molecules are highly stable, only the electrons with sufficient reduction potential can be utilized to trigger CO_2_ reduction reactions, and suitable photocatalyst is required to decrease the high reaction barrier. Among a wide range of metal and semiconductor photocatalysts for CO_2_ reduction, TiO_2_ materials has attracted much attention due to the advantageous of high reduction potential, low cost, and high stability. The activity, selectivity, and durability of TiO_2_ photocatalysts for CO_2_ reduction is related to the efficiency of electron-hole separation and light utilization ability, which are very sensitive to the surface structure, atomic configuration, and chemical composition of the photocatalysts. For example, different kinds of metals (transition, rare, alkali earth metals) have been studied as doping to improve the photocatalytic activity for CO_2_ reduction [92,93,94]. When compared to the metal doping method, which usually suffers from photocorrosion problem, non-metal (carbon, nitrogen, iodine, sulfur, etc.) doping has attracted more attention [95]. However, a large amount of non-intrinsic defects often generated during the doping and created electron-hole recombination centers at the same time. Herein, we mainly focus on surface/interface modification to enhance the performance of TiO_2_ photocatalysts towards CO_2_ reduction.

Yu et al. [64] investigated the effect of different exposed facets of anatase TiO_2_ crystals on the photocatalytic CO_2_ reduction activity. By using a simple fluorine-assisted hydrothermal method, they synthesized anatase TiO_2_ with different ratios of the exposed {101} and {001} facets. The results showed that the photocatalytic activity of the anatase TiO_2_ with the optimized ratio of exposed {001} to {101} facet (55:45) was ~4 times higher than that of P25 powder. They ascribed the enhancement to a concept of “surface heterojunction”. Electron and hole are driven to the {101} and {001} facets, inducing the seperation of electron and hole. It is worth mentioning that surface atomic and defect structures on different facets should also contribute the photocatalytic CO_2_ reduction processes. Truong et al. [96] synthesized rutile TiO_2_ nanocrystals with exposed high-index facets through solvothermal reaction by using a water-soluble titanium-glycolate complex as a precursor. Structural characterizations showed that each branched nanocrystal was bound by four facets of high-index {331} facets, and rutile {101} twinned structures were formed in the boundary of branches. The photocatalytic CO_2_ reduction to methanol showed a significantly higher activity was achieved in the synthesized nanostructures due to the abundant surface defects on the high energy facets.

Generating oxygen vacancies is effective to modulate the electronic/optical properties, and thus optimize diverse applications of metal oxides. Generally, bulk oxygen vacancies formed a middle sub-band in the forbidden gap, which made TiO_2_ response to the visible light, and those bulk oxygen vacancies also acted as the electron-hole recombination centers. The surface oxygen vacancies not only showed a strong response to the visible light, but also acted as the capture traps to inhibit electrons-holes recombination. By adjusting the concentration ratio of the surface and bulk oxygen vacancies, it is possible to improve the photocatalytic efficiency of TiO_2_ nanostructures. Li et al. [66] compared the effects of oxygen vacancies in TiO_2_ nanocrystals on the photoreduction of CO_2_. By choosing the precursors and post-treatment conditions, they obtained three kinds of TiO_2_ materials with different oxygen vacancies, i.e., TiO_2_ with surface oxygen vacancies (TiO_2_-SO), TiO_2_ with bulk single-electron-trapped oxygen vacancies (TiO_2_-BO), and TiO_2_ with mixed vacancies (TiO_2_-SBO). By analyzing the lifetime and intensity by positron annihilation, the efficiency of photocatalytic CO_2_ reduction improved with the increase of the ratio of surface oxygen vacancies to bulk ones. The results revealed the critical role of surface/bulk defects in photocatalytic properties. 

Similar to the case of photocatalytic hydrogen evolution, creating metal- or semiconductor- TiO_2_ interface via different post-deposition or in-situ forming methods has been demonstrated to be effective to improve the light harvesting and the separation of charged carriers, which are also important for the photoreduction of CO_2_. Specifically, Schottky barrier can be formed when the Fermi level of the deposited metals are lower than the conduction band of the TiO_2_ materials, which is favorable for the spatial separation of electron-hole pairs. Platinum, which possesses a suitable work function, is one of the most commonly used metal co-catalyst to improve the CO_2_ reduction performance of TiO_2_ photocatalysts. However, worldwide limited source and the consequent high price of platinum seriously hinder the large scale applications. The deposition of plasmonic nanostructures of metals such as silver and gold on TiO_2_ materials has been extensively studied due to the surface plasmon resonance (SPR) effect, which shows important role in improving the photocatalytic activity for CO_2_ reduction.

#### 4.1.4. Other Environmental Applications

The essence of antimicrobial by using TiO_2_ materials is a photocatalysis process. Therefore, the above surface/interface engineering towards photocatalytic enhancement can also be applied in the antimicrobial studies. Xu and co-workers [97] modified the aligned TiO_2_ nanotubes via a thin layer of graphitic C_3_N_4_ material by a chemical vapor deposition method. Due to the synergetic effect, the bactericidal efficiency against Escherichia coli irradiated by visible-light has been improved. Recently, self-cleaning materials have gained much attention in energy and environmental areas. The self-cleaning properties can be achieved by morphology design to form either hydrophilic or hydrophobic surfaces [98]. Previous works show that the hydrophilic or hydrophobic properties can be controlled by the photocatalytic process [99], making it possible to couple photocatalysis and photoinduced wettability to improve self-cleaning properties in a controllable way. TiO_2_ is such a material that shows photocalytic self-cleaning activity. Interface formation via heterojunction or heterostructure [100,101], surface modification [102], and elemental doping [103,104] are typical methods to improve photocatalytic and self-cleaning activities of TiO_2_ materials.

### 4.2. Lithium/Sodium Ion Batteries

Rechargeable lithium ion battery is one of the most important energy storage devices for a wide range of electron devices. The properties of electrode materials play an important role in the final performance of lithium ion batteries. Among the many potential electrode candidates, titanium dioxides with different phases have attracted much attention due to the abundance of raw materials and environmental benignity. Although the theoretical specific capacity of titanium dioxides (335 mA·h·g^−1^, based on the reaction TiO_2_ + *x*Li^+^ + *x*e^−^ ↔ Li*_x_*TiO_2_, *x*~ 0.96) is comparable to that of commercial graphite (372 mA·h·g^−1^), these materials possess a higher operating voltage platform than that of graphite, which is favorable for inhibiting the formation of lithium dendrite and solid-electrolyte interphase (SEI) layer. Moreover, the minor volume variation during cycling ensures a good cycling stability. It should be noted that the unsatisfied electronic conductivity and sluggish ion diffusion hinder the high-rate applications of these materials. The size, shape, composition, and assembly of TiO_2_ anodes are studied to optimize the lithium storage properties.

**Figure 6:**
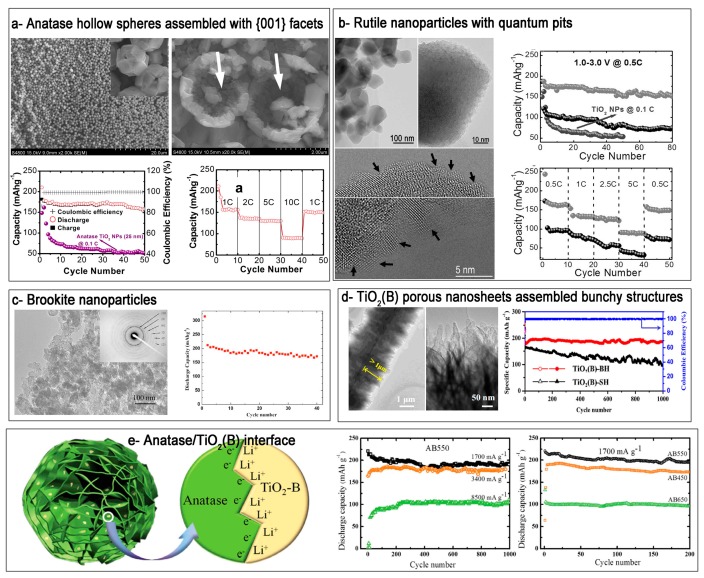
Typical TiO_2_ anodes and their lithium storage properties: (**a**) three-dimensional (3D) anatase TiO_2_ hollow microspheres assembled with high-energy {001} facets (reprinted from [105] with permission, Copyright The Royal Society of Chemistry, 2012); (**b**) Rutile TiO_2_ nanoparticles with quantum pits (reprinted from [50] with permission, Copyright The Royal Society of Chemistry, 2016); (**c**) Brookite TiO_2_ nanocrystalline (reprinted from [105] with permission, Copyright The Electrochemical Society, 2007); (**d**) bunchy hierarchical TiO_2_(B) structure assembled by porous nanosheets (reprinted from [119] with permission, Copyright Elsevier Ltd., 2017); and (**e**) Ultrathin anatase TiO_2_ nanosheets embedded with TiO_2_(B) nanodomains (Reproduced with permission from [125], Copyright John Wiley & Sons, 2015).

Recent works also show that nanoscale surface/interface design in TiO_2_ nanostructures is beneficial for improving the battery performance (Table 1), which are ascribed to the advantages of micro and nano architectures. For example, theoretical and experimental results demonstrated that lithium insertion was favored on the high-energy {001} facets in anatase phase, because of the open structure, as well as short path for ion diffusion. Since the first synthesis of anatase phase with exposed {001} facets by Yang et al. [43], extensive studies have been reported on the synthesis of TiO_2_ anodes with exposed {001} facets. Although the obtained anatase nanostructures possess sheetlike morphology exposed with {001} facets, the samples tend to over-lap to reduce the total surface energy. It is therefore important to prevent the aggregation of anatase nanosheets with exposed {001} facets. By using a simple one-pot solution method, we successfully obtained three-dimensional (3D) anatase TiO_2_ hollow microspheres, which were constituted by {001} facets (Figure 6a) [105]. In the synthesis, a mixture containing Ti powder, deionized water, hydrogen peroxide, and hydrofluoric acid was subjected to hydrothermal reaction at a temperature of 180 °C. The addition of hydrofluoric acid and hydrogen peroxide is critical for the formation of {001} facet assembly. The as-prepared sample shows good lithium storage properties. After 50 cycles at a current density of 0.1 C (1 C = 335 mA·h·g^−1^), a reversible capacity of 157 mA·h·g^−1^ can be retained, which is ~75% retention of the first reversible capacity. Rate performance test show that the discharge capacity reaches about 156 mA·h·g^−1^ after the first 10 cycles at the rate of 1 C, and then it slightly reduces to 135 and 130 mA·h·g^−1^ at the rates of 2 and 5 C, respectively. The electrode can still deliver a reversible capacity of 90 mA·h·g^−1^ even at a high rate of 10 C. The electrode resumes its original capacity of about 150 mA·h·g^−1^ after 10 cycles when the rate returns back to 1 C.

Rutile TiO_2_ is the most stable phase, which can be prepared at elevated temperatures, however, rutile TiO_2_ in bulk form is not favorable for the lithium ions intercalation. When the size decreased to nanoscale, rutile TiO_2_ phase possesses obvious activity towards the insertion of lithium ions even at room temperature. However, some critical problems should be considered when using rutile TiO_2_ nanostructures as anodes in lithium ion batteries, for example, particle aggregation and poor rate capacity. To boost the lithium storage of rutile TiO_2_ anodes, the synthesis of micro and nano configurations with optimized surface/interface and improved conductivity is an effective method to overcome the above limitations. We synthesized rutile TiO_2_ nanoparticles by a simple solution reaction, followed by annealing treatment (Figure 6b) [50]. The surface of each particle was etched to form quantum-sized pits (average size 2–5 nm), which possessed more unsaturated bond and other defect structures (for example steps, terraces, kinks, and others). The defective rutile TiO_2_ nanoparticles provided more active sites for the storage of lithium ions and improved the electron conductivity as well. As a consequence, the sample exhibited a specific capacity of ~145 mA·h·g^−1^ at a current density of 0.5 C with good rate capability (~102 mA·h·g^−1^ at 5 C) and cycling performance, demonstrating a great potential for lithium ion battery applications.

Among the different TiO_2_ polymorphs that were investigated, a severe capacity fading was noted for the brookite phase, although it exhibited nearly one mole of reversible lithium insertion/extraction in its nanostructured form [123]. There has not been extensive research focused on developing such an anode. Reddy et al. [124] demonstrated intercalation of lithium into brookite TiO_2_ nanoparticles (Figure 6c). Electrochemical test and ex-situ x-ray diffraction (XRD) studies showed that the structure was stable for lithium intercalation and deintercalation although the intercalation/deintercalation mechanism was not clear. Cycling performance of brookite TiO_2_ performed at C/10 rate in the voltage window 1.0–3.0 V showed that there is a gradual loss of capacity in the initial 10 cycles, and the capacity is fairly stable at 170 mA·h·g^−1^ on further cycling. In contrast to other TiO_2_ polymorphs, the TiO_2_(B) phase possesses relatively more open crystal structure, which allows for the facile insertion/extraction of lithium ions. Moreover, a lower operating potential (~1.55 V vs. Li) when compared to the anatase TiO_2_ (~1.75 V vs. Li), an improved reversibility, and a high rate capability make TiO_2_(B) phase a promising candidate for lithium storage. Li et al. [119] reported on the orderly integration of porous TiO_2_(B) nanosheets into bunchy hierarchical structure (TiO_2_(B)-BH) via a facile solvothermal process (Figure 6d). Benefiting from the unique structural merits, TiO_2_(B)-BH exhibited a high reversible capacity, long-term cycling stability (186.6 mA·h·g^−1^ at 1675 mA·g^−1^ after 1000 cycles), and a desirable rate performance.

Recently, Jamnik and Maier proposed that it was possible to store additional lithium at the interface of nanosized electrodes, which included solid–liquid (electrode-electrolyte) interface and solid–solid interface between the electrodes (Figure 6e) [125,134]. The interfaces can accommodate additional Li ions, leading to a rise of total Li storage. Meanwhile, an additional synergistic storage is favored if the electrode material is made of a lithium ion-accepting phase and an electron-accepting phase, which is beneficial for charge separation (“Job-sharing” mechanism). Along this line, Wu et al. [125] synthesized a new kind of microsphere that was constructed by ultrathin anatase nanosheets embedded with TiO_2_(B) nanodomains, which contained a large amount of interfaces between the two phases. The hierarchical nanostructures show capacities of 180 and 110 mA·h·g^−1^ after 1000 cycles at current densities of 3400 and 8500 mA·g^−1^. The ultrathin nanosheet structure, which provides short lithium diffusion length and high electrode/electrolyte contact area also accounts for the high capacity and long-cycle stability. This study highlights the importance of smart design in the interface structures in the nanoelectrodes.

Although the development and commercialization of lithium ion batteries have gained great success in the past years, one severe drawback of lithium ion batteries is the limited lithium resource in the Earth’s crust and its uneven geographical distribution. In this regard, sodium ion batteries have attracted particular attention due to the obvious advantages, including high earth-abundance of sodium, and lower cost vs. lithium ion batteries. In addition, the sodium chemistry is similar as the case of lithium, so the previously established surface/interface engineering strategies for titanium dioxides electrode design in lithium ion batteries system can be transferred to and expedite the sodium ion battery studies. Longoni et al. [39] systematically studied the role of different exposed crystal facets of the anatase nanocrystals on the sodium storage properties. By employing a surfactant-assisted solvothermal route, they synthesized anatase TiO_2_ nanostructures with three different morphologies (Rhombic elongated (RE), rhombic (R), and nanobar (NB)), which showed obvious differences in crystal face type exposition. Their electrochemical performance results, together with theoretical analysis, showed that an overcoordinated state of Ti atoms on the crystal surface (low energy density (101) facets of NB and R moieties) strongly inhibits the sodium uptake, while a Goldilocks condition seems to occur for crystalline faces with intermediate energy densities, like (100) in RE. Zhang et al. [131] reported a smart design of the assembly and interface of rutile TiO_2_, and fewer layer graphene by using carbon dots as designer additives. The resultant graphene-rich petal-like rutile TiO_2_ showed outstanding sodium-storage properties. At a rate of 0.25 C (83.75 mA·g^−1^) after 300 cycles, a high capacity of 245.3 mA·h·g^−1^ was obtained, even at a high current density of 12.5 C (4187.5 mA·g^−1^), a considerable capacity of 59.8 mA·h·g^−1^ can still be maintained. Notably, the reversible capacity up to 1100 cycles at a current density of 2.5 C (837.5 mA·g^−1^) can still reach 144.4 mA·h·g^−1^; even after 4000 cycles at 10 C (3350 mA·g^−1^), a capacity retention of as high as 94.4% is obtained. Zhang et al. [133] demonstrated the positive function of oxygen vacancies in TiO_2_(B) nanobelts for the enhancement of sodium storage. The sample displayed the significantly superior sodium-storage properties, including a higher capacity (0.5 C; 210.5 mA·h·g^−1^ vs. 102.7 mA·h·g^−1^), better rate performance (15 C; 89.8 vs. 36.7 mA·h·g^−1^), as compared to those of pristine TiO_2_(B) electrodes without oxygen vacancies.

### 4.3. Li–S Batteries

Li–S batteries possess exceptionally high theoretical energy densities ~2600 Wh·kg^−1^ vs. 580 Wh·kg^−1^ of today’s best batteries. Li–S batteries contain low cost materials, sulfur is highly abundant, and the anode consists of lithium metal and does not limit the capacity. Today’s Li–S technology falls short in energy density and lifetime because of the limited sulfur loading in the cathode, due to the poor conductivity of sulfur deposits, because of the solvation into the electrolyte of the discharge products (i.e., Li*_x_*S*_y_* polysulfides), and finally because of the large volume expansion of sulfur during the battery cycling affecting the cathode integrity.

Cathodes with high surface area and high electronic conductivity are crucial to improve sulfur loading and rate performance of Li–S batteries. The polysulfides “shuttle” phenomena, via the solvation of the polysulfides in the electrolyte, gradually decrease the mass of active material, leading to continuous fading in capacity and must be avoided. Therefore, the candidate cathodes should have a porous and conductive nature, as well as suitable interactions with polysulfides simultaneously. To overcome those obstacles, a wide range of strategies has been developed, including encapsulation or coating of the sulfur electrode, use of impermeable membranes, and/or the use of electrolytes that minimize the solubility and diffusivity of the polysulfides. However, none of these solutions has led to acceptable results, fulfilling all of the requirements. For example, the main disadvantage of widely used porous conductive carbon electrodes lies in weak physical confinement of lithium sulfides, which is insufficient to prevent the diffusion and shuttling of polysulfides during long-term cycling. Therefore, ideal electrodes should not only possess porous and conductive nature, but also suitable interactions with polysulfides.

**Figure 7:**
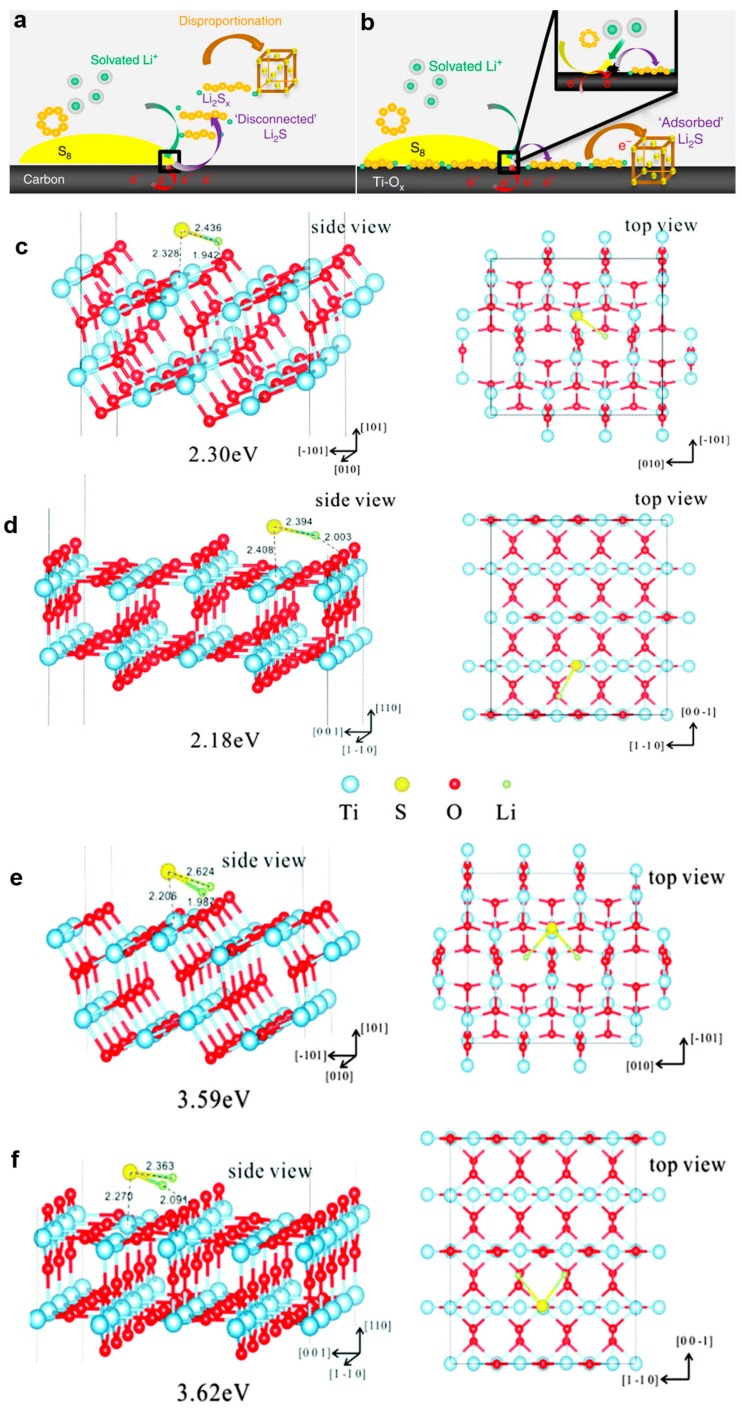
The interaction between sulfur or lithium polysulphides and electrodes. (**a**) On reduction of S_8_ on a carbon host, Li_2_S*_X_* desorb from the surface and undergo solution-mediated reactions leading to broadly distributed precipitation of Li_2_S; (**b**) On reduction of S_8_ on the metallic polar Ti_4_O_7_, Li_2_S*_X_* adsorb on the surface and are reduced to Li_2_S via surface-mediated reduction at the interface (reprinted from [135] with permission, Copyright Nature Publishing Group, 2014); Adsorption configuration of (**c**,**d**) Li–S* and (**e**,**f**) Li_2_S on the (**c**,**e**) anatase-TiO_2_ (101) surface and (**d**,**f**) rutile-TiO_2_ (110) surface (Reproduced with permission from [136], Copyright The Royal Society of Chemistry, 2016).

On a typical carbon support (Figure 7a), elemental sulfur undergoes reduction to form lithium polysulphides that then dissolve into the electrolyte. In the presence of a polar metal oxide as witnessed for titanium oxides, however, the solvation of the polysulfides is significantly affected (Figure 7b). Not only is the concentration of polysulphides in solution that greatly diminished during discharge, but also a slow, controlled deposition of Li_2_S is observed. The results are ascribed to the interface-mediated, spatially controlled reduction of the polysulphides. Yu et al. [136] studied the interactions between intermediate polysulphides, final discharge product Li_2_S and stable TiO_2_ surface (anatase-TiO_2_ (101), rutile-TiO_2_ (110)) via theoretical simulation (Figure 7c–f). Their results show that the binding strength of the polysulphides to the anatase-TiO_2_ (101) surface (2.30 eV) is a little higher than to rutile-TiO_2_ (110) surface (2.18 eV), and the binding energy of Li_2_S to the anatase-TiO_2_ (101) surface (3.59 eV) is almost the same as with the rutile-TiO_2_ (110) surface (3.62 eV). The values are larger than the adsorption binding energies for Li–S composites on graphene (<1 eV), highlighting the efficacy of TiO_2_ in binding with polysulfide anions via polar–polar interactions.

**Figure 8:**
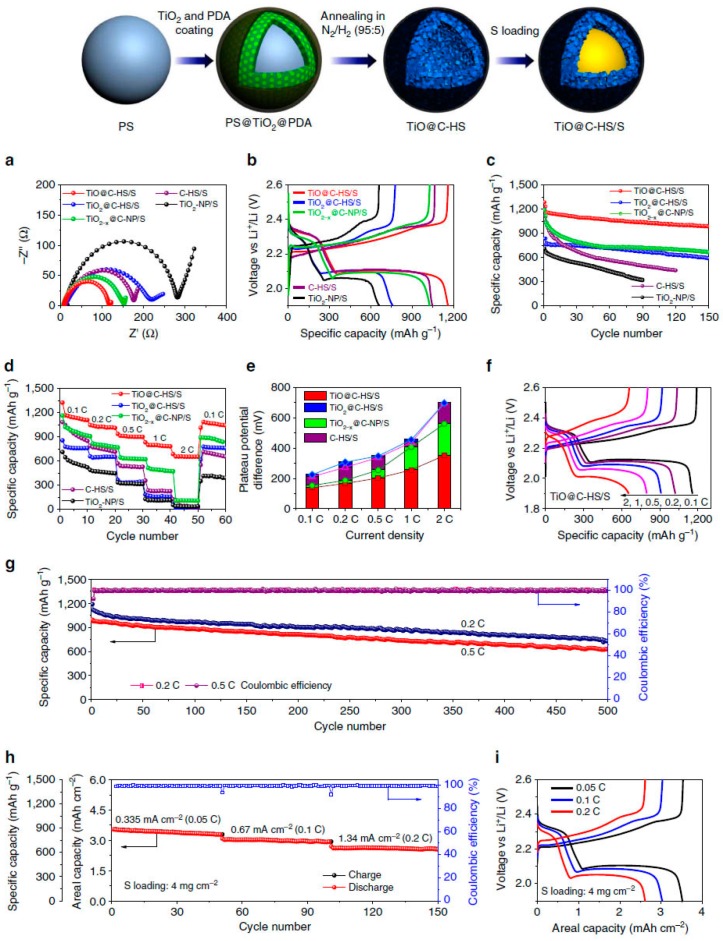
Schematic illustration of the synthesis process and electrochemical properties of TiO@C-HS/S composites. (**a**) Nyquist plots before cycling from 1 MHz to 100 mHz; (**b**) the second-cycle galvanostatic charge/discharge voltage profiles at 0.1 C; (**c**) cycle performances at 0.1 C; (**d**) rate capabilities; and (**e**) the potential differences between the charge and discharge plateaus at various current densities of the TiO@C-HS/S, titanium dioxide@carbon hollow nanospheres/S composite (TiO_2_@C-HS/S), carbon coated conductive TiO_2-*x*_ nanoparticles/S composite (TiO_2-*x*_@C-NP/S), pure carbon hollow spheres/S composite (C-HS/S) and TiO_2_ nanoparticles/S composite (TiO_2_-NP/S) electrodes. (**f**) Voltage profiles at various current densities from 0.1 to 2 C and (**g**) prolonged cycle life and Coulombic efficiency at 0.2 and 0.5C of the TiO@C-HS/S electrode. (**h**) Areal capacities and (**i**) voltage profiles at various current densities from 0.335 (0.05 C) to 1.34 mA·cm^−2^ (0.2 C) of the TiO@C-HS/S electrode with high sulfur mass loading of 4.0 mg·cm^−2^ (reprinted from [40] with permission, Copyright Nature Publishing Group, 2016).

Experimentally, Cui et al. [137] designed a unique sulfur-TiO_2_ yolk-shell architecture as a sulfur cathode, and obtained an initial specific capacity of 1030 mA·h·g^−1^ at 0.5 C (1 C = 1673 mA·g^−1^) and Coulombic efficiency of 98.4% over 1000 cycles. Impressively, the capacity decay at the end of 1000 cycles is found to be as small as 0.033% per cycle (3.3% per 100 cycles). The excellent properties were ascribed to the yolk–shell morphology, which accommodates the large volumetric expansion of sulfur during cycling, thus preserving the structural integrity of the shell to minimize polysulphide dissolution. Based on the knowledge of chemical interactions between polysulphides and titanium oxides, a wide range of methods have been performed to optimize configuration of sulfur-titanium oxide cathodes. Typical examples include design and synthesis of porous titanium oxides high-surface area, crystalline facts engineering, conductivity enhancement by adding conductive agents (such as carbon fibers, graphene, conductive polymers) into the titanium oxide nanostructures or through annealing in inert/H_2_ atmosphere. In this regard, Lou et al. [40] synthesized a sulfur host containing titanium monoxide@carbon hollow nanospheres (TiO@C-HS/S), which possess the key structural elements (i.e., high surface area, conductive, interactions with polysulfides) that are required for high-performance cathodes simultaneously (Figure 8). The TiO@C/S composite cathode delivered high discharge capacities of 41,100 mA·h·g^−1^ at 0.1 C, and exhibited stable cycle life up to 500 cycles at 0.2 and 0.5 C with a small capacity decay rate of 0.08% per cycle. The Li–S batteries performance based on typical titanium oxides are summarized in Table 2.

## 5. Phase Stability of TiO_2_ Nanostructures 

As a kind of chemically stable and environmentally compatible metal oxides, TiO_2_ nanostructures show fantastic physical/chemical properties and find many practical applications, ranging from energy conversion and storage, as mentioned above and others. The properties and applications are determined by the structures of TiO_2_ materials, which is related to the external (temperature, pressure, environment, etc.) and internal (composition, stain, etc.) factors. Overall, the relative phase stability in ambient bulk form is TiO_2_(B) < anatase < brookite < rutile, and the specific phase shows its unique applications. For example, anatase has been found to be the most active phase in photocatalysis. TiO_2_(B) phase is more favorable for the insertion/extraction of lithium ions due to the more open crystal structure when compared to the other TiO_2_ phases. Therefore, it is of importance to understand the phase transformation on nanoscale and improve the phase stability of the related TiO_2_ nanostructures. General thermodynamic investigation, computational methods (including molecular dynamics simulations and DFT calculations), experimental routes (XRD, calorimetry, electrochemical measurements, etc.) have been successfully employed to study the phase stability and coarsening kinetics of the typical TiO_2_ phases under different environment (dry, wet, hydrothermal conditions) [146]. Several excellent reviews describing the topics are available elsewhere, and we do not discuss them in this paper. 

With the decreasing of the size or dimension, surface and/or interface will dominate in the nanostructure and play an important role in phase stability. Due to the nature of coordination unsaturation, the atoms at the surface are more active than those within the interior. Therefore, surfaces usually exhibit a lower stability relative to the lattice interior part. For example, the melting point of free-standing nanoparticles is remarkably depressed relative to that of bulk phase (*T*_0_). Stabilizing the surface atoms would be a way to improve the relative phase stability. Typically, when nanoparticles are properly coated by or embedded in a matrix with higher melting point, the melting point of the particles can be elevated above *T*_0_. Herein, we focus on the strategies of surface/interface engineering to tune the phase stability in typical TiO_2_ nanostructures. 

**Figure 9:**
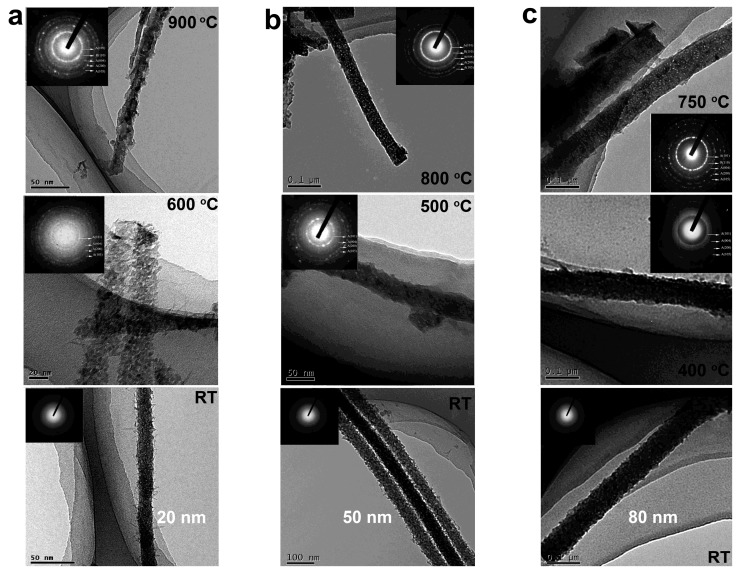
Typical transmission electron microscopy (TEM) images of the as-prepared and annealed TiO_2_ nanowires with diameters of (**a**) 20; (**b**) 50; and (**c**) 80 nm. The insets show corresponding selected area electron diffraction (SAED) patterns (Reproduced with permission from [16], Copyright Springer, 2012).

We systematically studied the crystallization and structural transformation from anatase to rutile phase in the initial amorphous TiO_2_ nanowires embedded in anodic aluminum oxide with different diameters (20, 50, and 80 nm, termed as TiO_2_-20, TiO_2_-50, and TiO_2_-80, hereafter) [16]. Electron microscopy analysis and XRD results showed that the crystallization of TiO_2_-20, TiO_2_-50, and TiO_2_-80 from amorphous to anatase occurred at ~600, ~500, and ~400 °C, and the transformation from anatase to rutile phase started at ~900, ~800, and ~750 °C (Figure 9). The results revealed a strong size dependence of the thermal stability of TiO_2_ nanowires embedded the template. Control experiments on amorphous TiO_2_ powder showed the crystallization and phase transformation temperatures were ~200 and ~600 °C, respectively. 

**Figure 10:**
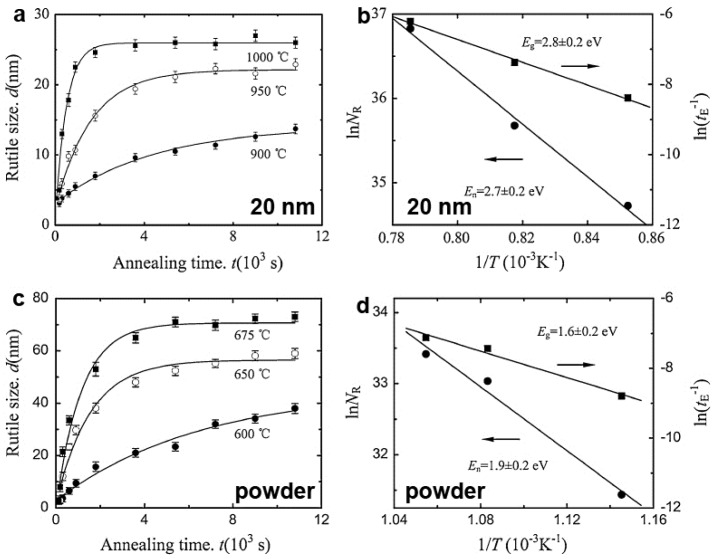
Nucleation and growth kinetics of nanocrystalline anatase to rutile. Annealing time dependence of the size of the rutile in the (**a**) nanowire and (**c**) free-state powders at different temperatures; Annealing temperature variations of the nucleation rate (NR) and the growth saturation rate *t*_E_^−1^ for rutile in the (**b**) nanowire and (**d**) free-state powders, respectively (Reproduced with permission from [16], Copyright Springer, 2012).

To quantitatively study the nucleation and growth kinetics, in-situ high-temperature X-ray diffraction technique was employed to track the transformation process from anatase to rutile phase. In this method, the position and intensity of diffraction peaks change during the increasing and decreasing temperatures, and thus provide an effective and direct way to trace the phase structure. Taken TiO_2_-20 and TiO_2_ powder for typical examples, the transformed rutile phase showed an exponential growth versus annealing time *t*, and the growth of the rutile was a thermally activated process (Figure 10). The rutile growth activation energy (*E_g_*) values of 2.8 ± 0.2 eV and 1.6 ± 0.2 eV were determined in TiO_2_-20 and TiO_2_ power, respectively. Additionally, no obvious change of the rutile size was observed in the initial stage of the studied temperature range, indicating that the increasing of the rutile volume fraction was induced by the nucleation events. By analyzing the dependence of nucleation rate on the annealing temperature, the rutile nucleation activation energy (*E_n_*) values of 2.7 ± 0.2 eV and 1.9 ± 0.2 eV were yielded for TiO_2_-20 and TiO_2_ power, respectively. The higher nucleation and growth energy for TiO_2_-20 implied that the phase transformation from anatase to rutile was inhibited, i.e. the thermal stability of the anatase phase was improved. Our theoretical work showed the difference of thermal expansion coefficient between the nanoscale channel (aluminum oxide) and the embedded TiO_2_ nanowire generated overpressure on the TiO_2_/Al_2_O_3_ interface during annealing. The pressure can be estimated as ~0.13 GPa at 900 °C for TiO_2_-20 sample. The pressure compressed the anatase surface and constrained the vibration of surface atoms, which were responsible for the improvement of the anatase phase. By choosing suitable surface layers and other coating techniques (such as Langmuir-Blodgett assembly, atomic layer deposition, etc.), this surface/interface confinement strategy can also be used to improve the phase stability of other TiO_2_ polymorphs. For example, Zazpe et al. [15] recently reported on a very obvious enhancement of the phase stability of selforganized TiO_2_ nanotubes layers with amorphous structure, which are provided by thin Al_2_O_3_ coatings of different thicknesses prepared by atomic layer deposition. TiO_2_ nanotube layers coated with Al_2_O_3_ coatings exhibit significantly improved thermal stability, as illustrated by the preservation of the nanotubular structure upon annealing treatment at high temperatures (870 °C). It is worth noting that accompanying by phase transformation during annealing, TiO_2_ nanostructures also suffer from the change in size, surface area, bandgap, and morphology [147], which are important parameters that influence the applications and must be considered in the phase stability studies. 

**Figure 11:**
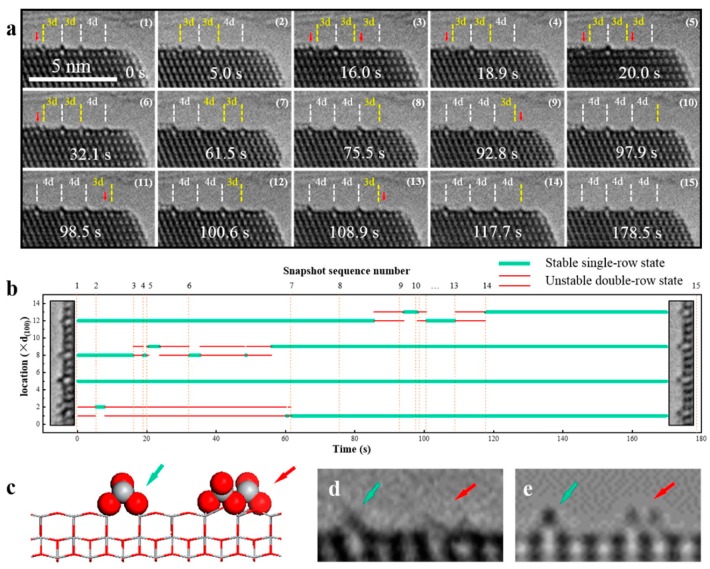
Atomic evolution of the (1 × *n*) reconstructions on anatase TiO_2_ (001) surface. (**a**) Sequential HRTEM images of the dynamic structural evolution, viewed from [010] direction, with the red arrows indicating the unstable states; (**b**) The statistical diagram of the locations of the TiO*_x_* rows with green and red lines indicating the stable states and the unstable states; (**c**) Side view of the proposed model for the unstable two-row state with the TiO*_x_* row shown as ball-and-stick (Ti, gray; O, red) on the TiO_2_ stick framework. The green and red arrows indicate the stable single-row and instable double-row structures, respectively; (**d**,**e**) Experimental HRTEM image compared with the simulated image based on the model in (**c**). (Reproduced with permission from [149], American Chemical Society, 2016).

Besides phase transformation among the different TiO_2_ polymorphs, surface atomic rearrangement (reconstruction) also occurs to reach a more stable state at a certain environment (temperature, pressure, atmosphere, humidity, etc.). Remarkably different physical/chemical properties on the surface with respect to the bulk counterpart can be yielded by the reconstruction. The environmental transmission electron microscopy (ETEM) technique allows for the direct imaging of the samples that are placed in a specimen chamber that is high pressures attainable, which can be achieved by either differential pumping systems or delicate TEM holder design [148]. Yuan et al. [149] reported in-situ atomic scale ETEM observations of the formation and evolution of the (1 × 4) reconstruction dynamics on the anatase TiO_2_ (001) surface under oxygen atmosphere. They firstly cleaned the wet chemistry synthesized TiO_2_ nanosheets with the aid of e-beam irradiation at a temperature of 500 °C in oxygen environment. On the cleaned TiO_2_ surface, the real-time dynamics for the transition from metastable (1 × 3) and (1 × 5) to (1 × 4), and the unstable intermediate states were observed and identified (Figure 11). The special reconstruction was driven by the lowly coordinated atoms and surface stress. The results demonstrate the power of in situ real-time technique to study the dynamic formation and evolution of surface structures.

## 6. Conclusions and Perspective

Recent years have witnessed explosive research and development efforts on TiO_2_ materials, ranging from controllable synthesis to advanced characterizations and device applications. Although the intrinsic properties, such as wide bandgap, rapid carriers recombination, poor electronic conductivity, and coexistence of multiphases, hampered the practical applications of pristine TiO_2_ materials to some extent, the surface/interface modifications have been demonstrated as effective routes to break the limitations, making it possible to be applied in diverse areas. This review article summarized the main progress in engineering the surface/interface structures in TiO_2_ micro and nano structures, discussed the effect of surface/interface structures on environmental and electrochemical applications. Specifically, by tuning the exposed crystallographic planes, engineering defect structures, and constructing interface in various TiO_2_ materials, the heterogeneous photocatalysis process, including light absorption, the generation and separation of photoexcited carriers, the migration, transport and recombination of carriers, and surface catalytic reactions can be well controlled and optimized. As a result, the photocatalytic properties of TiO_2_ materials in the degradation of organic contaminants, hydrogen evolution, CO_2_ reduction, antimicrobial, and self-cleaning are greatly improved. For the battery applications, engineering the surface/interface structures of TiO_2_ crystal not only increase the sites for ion storage, but also improve the electron and ion conductivity. In Li–S battery system, the interaction between sulfur cathodes and the surface of TiO_2_ host can also be adjusted by surface/interface engineering. All of those factors are crucial for improving the specific capacity, rate performance, and cycle durability. In addition, the phase transitions in TiO_2_ nanostructures and possible strategies of improving the phase stability have been analyzed. Despite these impressive advances, several challenges still remain.

(1) Developing novel synthesis and treatment methods. Despite great success has been obtained in the controllable synthesis of TiO_2_ nanostructures with tailored micro and nano structures, there is still room for improvement in terms of quality of the products. Moreover, the new methods also provide opportunities to further understand the nucleation and growth.
(2) Control of the fine structures. High-index facets and defect sites are chemically active. However, the synthesis of TiO_2_ nanocrystals with specific high-index facets is still a challenge. It is highly desirable to synthesize facet-controllable TiO_2_ materials and further study the facet effect on energy storage, conversion, and other applications. In addition, selectively generating defect structures and controlling their concentrations in different TiO_2_ phases are significant to revel the role of defects in various physical and chemical processes.
(3) In situ/*operando* study the dynamic evolution of the surface/interface. In situ*/operando* spectroscopic or microscopic studies afford the chance to probe the evolution of TiO_2_ surface/interface structures in working conditions, which is crucial to study the complex phase transformation and device stability.

## Figures and Tables

**Table 1:** Performance comparison of some lithium ion batteries and sodium ion batteries based on typical titanium dioxide (TiO_2_) anodes (the voltage is versus Li^+^/Li or Na^+^/Na).

Material/[Reference]	Capacity (Cycles) (mA·h·g^−1^)	Rate Capability (mA·h·g^−1^)	Voltage (V)
Rutile TiO_2_ with quantum pits [50]	145 (80)@168 mA·g^−1^	102@1675 mA·g^−1^	1–3/Li
TiO_2_ microboxes [106]	187 (300)@170 mA·g^−1^	63@3400 mA·g^−1^	1–3/Li
Rutile TiO_2_ inverse opals [107]	95 (5000)@450 mA·g^−1^	-	1–3/Li
Faceted TiO_2_ crystals [108]	141.2 (100)@170 mA·g^−1^	29.9@1700 mA·g^−1^	1–3/Li
Nanosheet-constructed TiO_2_(B) [109]	200 (200)@3350 mA·g^−1^	216@3350 mA·g^−1^	1–3/Li
TiO_2_ hollow microspheres [105]	157 (50)@170 mA·g^−1^	90@1700 mA·g^−1^	1–3/Li
rutile TiO_2_ nanostructures [110]	190 (200)@102 mA·g^−1^	84.5@1700 mA·g^−1^	1–3/Li
nest-like TiO_2_ hollow microspheres [111]	152 (100)@1020 mA·g^−1^	130@3400 mA·g^−1^	1–3/Li
Co_3_O_4_ NPs@TiO_2_(B) NSs [112]	677.3 (80)@100 mA·g^−1^	386@1000 mA·g^−1^	0.01–3.0/Li
TiO_2_(B)@VS_2_ nanowire arrays [113]	365.4 (500)@335 mA·g^−1^	171.2@3350 mA·g^−1^	0.01–3.0/Li
Nb-doped rutile TiO_2_ Mesocrystals [114]	141.9 (600)@850 mA·g^−1^	96.3@6800 mA·g^−1^	1–3/Li
TiO_2_@defect-rich MoS_2_ nanosheets [115]	805.3 (100)@100 mA·g^−1^	507.6@2000 mA·g^−1^	0.005–3.0/Li
MoS_2_-TiO_2_ based composites [116]	648 (400)@1000 mA·g^−1^	511@2000 mA·g^−1^	0.005–3.0/Li
macroporous TiO_2_ [117]	181 (1000)@1700 mA·g^−1^	69@12.5 A·g^−1^	1–3/Li
porous TiO_2_ hollow microspheres [118]	216 (100)@170 mA·g^−1^	112@1700 mA·g^−1^	1–3/Li
porous TiO_2_(B) nanosheets [119]	186 (1000)@1675 mA·g^−1^	159@6700 mA·g^−1^	1–3/Li
graphene supported TiO_2_(B) sheets [120]	325 (10000)@500 mA·g^−1^	49@40 A·g^−1^	1–3/Li
mesoporous TiO_2_ coating on carbon [121]	210 (1000)@3400 mA·g^−1^	150@10.2 A·g^−1^	1–3/Li
Ti^3+^-free three-phase Li_4_Ti_5_O_12_/TiO_2_ [122]	136 (1000)@4000 mA·g^−1^	155.6@8 A·g^−1^	1.0–2.5/Li
Mesoporous TiO_2_ [123]	149 (100)@1000 mA·g^−1^	104@2000 mA·g^−1^	1–3/Li
Nanocrystalline brookite TiO_2_ [124]	170 (40)@35 mA·g^−1^	-	1–3/Li
Anatase TiO_2_ embedded with TiO_2_(B) [125]	190 (1000)@1700 mA·g^−1^	110@8500 mA·g^−1^	1–3/Li
TiO_2_-Sn@carbon nanofibers [126]	413 (400)@100 mA·g^−1^	-	0.01–2.0/Na
Double-walled Sb@TiO_2-*x*_ nanotubes [127]	300 (1000)@2.64 A·g^−1^	312@13.2 A·g^−1^	0.1–2.5/Na
Carbon-coated anatase TiO_2_ [128]	180 (500)@1675 mA·g^−1^	134@3.35 A·g^−1^	0.05–2.0/Na
Nanotube arrays of S-doped TiO_2_ [129]	136 (4400)@3350 mA·g^−1^	167@3350 mA·g^−1^	0.1–2.5/Na
Amorphous TiO_2_ inverse opal [130]	203 (100)@100 mA·g^−1^	113@5 A·g^−1^	0.01–3.0/Na
Petal-like rutile TiO_2_ [131]	144.4 (1100)@837.5 mA·g^−1^	59.8@4187 mA·g^−1^	0.01–3.0/Na
Yolk-like TiO_2_ [132]	200.7 (550)@335 mA·g^−1^	90.6@8375 mA·g^−1^	0.01–3.0/Na
Blue TiO_2_(B) nanobelts [133]	210.5 (5000)@3350 mA·g^−1^	90.6@5025 mA·g^−1^	0.01–3.0/Na

**Table 2:** Comparison of Li–S batteries performance based on typical titanium oxides electrode (the voltage is versus Li^+^/Li).

Material/[Reference]	Capacity (Cycles) (mA·h·g^−1^)	Rate Capability (mA·h·g^−1^)	Sulfur Loading (%)	Voltage (V)
TiO@carbon [40]	750 (500)@335 mA·g^−1^	655 @3.35 A·g^−1^	~70	1.9–2.6
Ti_4_O_7_/S [135]	1070 (500)@3350 mA·g^−1^	-	70	1.8–3.0
TiO_2_/N-doped graphene [136]	918 (500)@1675 mA·g^−1^	833 @6.7 A·g^−1^	59	1.7–2.8
S–TiO_2_ yolk–shell [137]	1030 (1000)@837 mA·g^−1^	630 @3.35 A·g^−1^	62	1.7–2.6
TiO_2_-porous carbon nanofibers [138]	618 (500)@1675 mA·g^−1^	668 @8.375 A·g^−1^	55	1.7–2.6
TiO_2_-carbon nanofibers [139]	694 (500)@1675 mA·g^−1^	540 @3.35 mA·g^−1^	68.83	1.7–2.8
TiO_2_/graphene [140]	630 (1000)@3350 mA·g^−1^	535 @5.025 A·g^−1^	51.2	1.6–2.8
Porous Ti_4_O_7_ particles [141]	989 (300)@167.5 mA·g^−1^	873 @1.675 A·g^−1^	50-55	1.8–3.0
Polypyrrole/TiO_2_ nanotube arrays [142]	1150 (100)@167.5 mA·g^−1^	-	61.93	1.8–3.0
Graphene-TiO_2_ NPs [143]	663 (100)@1675 mA·g^−1^	-	75	1.7–2.8
TiO_2_ nanowire/graphene [144]	1053 (200)@335 mA·g^−1^	-	60	1.5–2.8
graphene/TiO_2_/S [145]	597 (100)@1675 mA·g^−1^	-	60	1.5–3.0
